# Chemodiversity and Bioactivity of the Essential Oils of *Juniperus* and Implication for Taxonomy

**DOI:** 10.3390/ijms242015203

**Published:** 2023-10-15

**Authors:** Huizhong Hu, Dengwu Li, Ruxue Bai, Weiping Zhang, Hong Luo, Enping Yu

**Affiliations:** 1 College of Forestry, Northwest A & F University, Yangling 712100, China; hhz1811@163.com (H.H.); bgptwtd@163.com (R.B.); xncuimin@nwafu.edu.cn (W.Z.); 18628332921@163.com (H.L.); yuenp@scbg.ac.cn (E.Y.); 2 Shaanxi Key Laboratory of Economic Plant Resources Development and Utilization, Yangling 712100, China

**Keywords:** chemical composition, α-diversity, antioxidant activity, antibacterial activity, key compounds

## Abstract

The essential oils of *Juniperus* are highly beneficial medicinally. The present study aimed to assess the chemodiversity and bioactivity of *Juniperus formosana*, *Juniperus przewalskii*, *Juniperus convallium*, *Juniperus tibetica*, *Juniperus komarovii*, and *Juniperus sabina* essential oils from the Qinghai-Tibet Plateau. The results revealed 92 components in six essential oils: α-pinene (2.71–17.31%), sabinene (4.91–19.83%), and sylvestrene (1.84–8.58%) were the main components. Twelve components were firstly reported in *Juniperus* oils, indicating that the geographical location and climatic conditions of the Qinghai-Tibet Plateau produced the unique characteristics of *Juniperus* essential oils. The chemodiversity of *Juniperus* essential oils varied greatly, with *J. sabina* having the most recognized components (**64**) and the highest chemodiversity (Shannon–Wiener index of 3.07, Simpson’s diversity index of 0.91, and Pielou evenness of 0.74). According to the chemodiversity of essential oils, the six plants were decided into the α-pinene chemotype (*J. formosana*), hedycaryol chemotype (*J. przewalskii*, *J. komarovii*, *J. convallium*, *J. tibetica*), and sabinene chemotype (*J. sabina*). PCA, HCA and OPLS-DA showed that *J. formosana* and *J. sabina* were distantly related to other plants, which provides a chemical basis for the classification of *Juniperus* plants. Furthermore, bioactivity tests exhibited certain antioxidant and antibacterial effects in six *Juniperus oils*. And the bioactivities of *J. convallium*, *J. tibetica*, and *J. komarovvii* were measured for the first time, broadening the range of applications of *Juniperus*. Correlation analysis of components and bioactivities showed that δ-amorphene, β-udesmol, α-muurolol, and 2-nonanone performed well in the determination of antioxidant activity, and α-pinene, camphene, β-myrcene, as well as (E)-thujone, had strong inhibitory effects on pathogenic bacteria, providing a theoretical basis for further research on these components.

## 1. Introduction

Essential oils (EOs), also known as volatile oils, are mixtures of secondary metabolites produced by aromatic plants [1]. In recent years, EOs have been found to exhibit antibacterial, anti-inflammatory, analgesic, and antioxidant effects [2,3], which has sparked an increasing interest in plant EOs.

Essential oils are widely distributed in plants. As the second largest conifer genus, most *Juniperus* plants are rich in EOs [4,5]. The principal ingredients of the aromatic oils from *Juniperus* are α-pinene, β-pinene, limonene, sabinene, myrcene, dl-limonene, bornyl acetate, and other compounds [6,7]. These secondary metabolites have a good biological activity, enabling EOs from *Juniperus* to be utilized extensively in different regions [8,9,10,11,12]. Because of its remarkable adaptability, *Juniperus* has a wide geographic distribution from the Arctic Circle to the highlands of the African tropics. Thus, there are certain differences in EOs among various species and geographical regions [13,14].

The highest plateau in the world is the Qinghai-Tibet Plateau, with an average elevation of 4500 m [15]. In total, 18 *Juniperus* species, including *Juniperus formosana* Hayata, *J. przewalskii* Komarov, *J. convallium* Rehder & E. H. Wilson, *J. tibetica* Komarov, *J. komarovii* Florin, *J. sabina* L., all have a sizable number in the plateau [16]. With a broad distribution in the adret and semi-adret, *Juniperus* has become a dominant genus in the forest ecosystem and diversity of the Qinghai-Tibet Plateau. Little variations in altitude and climate may have an impact on the volatile compounds and activity [17,18], therefore we hypothesized ① the highland environment created the specificity of EOs from *Juniperus*; ② the chemodiversity of essential oils was consistent with plant taxonomy; ③ six juniper essential oils had antioxidant and bacteriostatic activities.

In this study, steam distillation and GC-MS were used to separate and identify the EOs from the six common species of *J. formosana*, *J. przewalskii*, *J. convallium*, *J. tibetica*, *J. komarovii*, and *J. sabina* on the Qinghai-Tibet Plateau to analyze the specificity and diversity of the *Juniperus* phytochemical composition in this region. Their antioxidant and antibacterial activities were determined, providing resources for the utilization of *Juniperus* and high-quality germplasm breeding.

## 2. Results

### 2.1. Chemodiversity of Juniperus Essential Oils

#### 2.1.1. Essential Oils Yields and Composition

The yields and composition of EOs from different species are shown in Table 1. The EOs yields of the six *Juniperus* plants ranged from 1.30 to 4.13% with an average yield of 2.63%. *J. convallium* (4.13%) and *J. przewalskii* (3.40%) produced the highest yield. GC-MS analysis found 92 compounds, including monoterpene hydrocarbons (**13**), oxygenated monoterpenes (**34**), sesquiterpene hydrocarbons (**16**), oxygenated sesquiterpenes (**18**), and other substances (**11**) accounting for 58.35–85.04% of the total components. Monoterpene hydrocarbons were the main volatile components in the EOs, with an average level of 35.22%.

**Figure 1:**
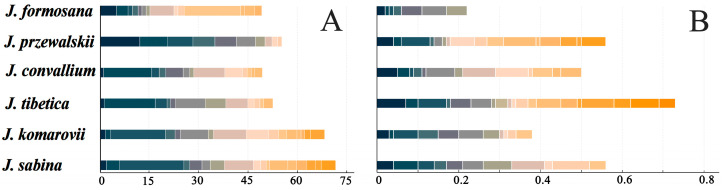
Main components (**A**) and trace components (**B**) of essential oils from six *Juniperus* species. Different colors represent different compounds.

In this study, *J. sabina* had the most main components (more than 1% of the total), with a total of 17 species up to 72.61% (Figure 1A). Among them, sabinene had the highest content of 19.83%. In addition to being one of the main constituents of *J. sabina*, sabinene was also the most prevalent element in the EOs of *J. przewalskii*, *J. convallium*, *J. tibetica*, and *J. komarovii*, and its percentages varied widely between these populations (12.14–19.83%). In the oil of *J. formosana*, α-pinene dominated with the highest content (17.31%) among the 14 main components. *J. prizewalskii* found the least main components (**10**), with a total content of 55.91%.

As illustrated in Figure 1B, 40 trace components, making up less than 0.1% of the entire composition, were discovered and represented in an average of 0.52% of the total oil content. Nineteen trace components from *J. tibetica* had a total quantity of 0.73, making them the most prevalent. In terms of trace components, *J. przewalskii* was second only to *J. tibetica* in quantity and content. Notably, the minimal seven trace components were found from *J. formosana* EOs.

#### 2.1.2. Shared and Unique Components

**Figure 2:**
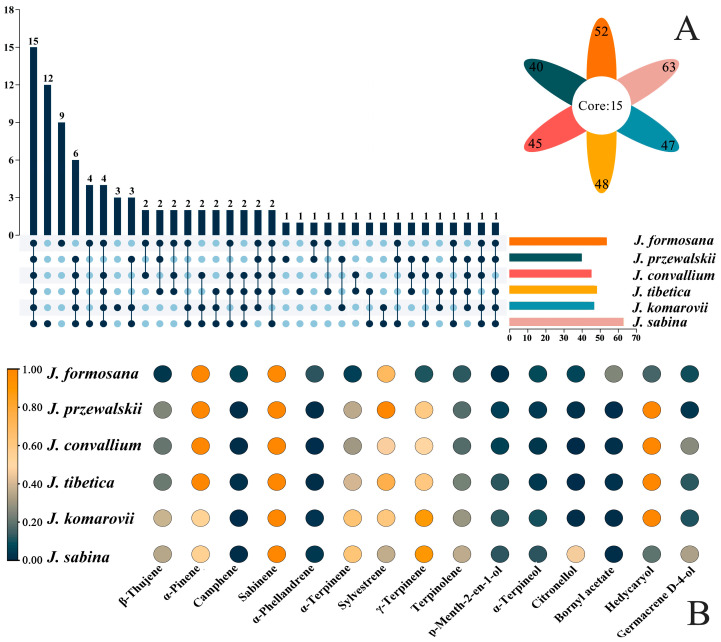
Distributions of shared and unique components (**A**) and correlation analysis of common compounds (**B**) in essential oils from six Juniperus species. Different numbers indicate the number of compound species, and the connecting lines indicate that these species share compounds (**A**). Different colors represent the relative contents of compounds (**B**).

Upset analysis was performed to visualize the distributions of the six EOs’ common and unique components (Figure 2A). The findings revealed that six EOs shared 15 components, including β-thujene, α-pinene, camphene, sabinene, α-phellandrene, α-terpinene, etc. (Figure 2B). The total content of shared components in the six species has fluctuated from a minimum of 31.64% to a maximum of 45.21%. Among them, α-pinene, sabinene, and sylvestrene showed the highest amounts in the six EOs. Figure 2B displays the heatmap of 15 common components, showing the close interconnectedness among *Juniperus* species and the main components. The higher levels of shared components, α-pinene, sabinene, and sylvestrene, showed strong correlations.

*J. sabina* and *J. formosana* have more distinctive parts (Figure 2A). Twelve different compounds, including 2-undecanone, α-cadinol, (E)-geranic acid methyl ester, 1-epi-cubenol, etc., were combined in *J. sabina* EOs with a total of 7.44% (Table 1). A total of nine unique ingredients were distributed in *J. formosana* essential oil, accounting for 3.78% of the total content. *J. przewalskii* and *J. tibetica* had only one unique ingredient, while no unique ingredients existed in *J. convallium* EOs.

#### 2.1.3. α-Diversity of *Juniperus* Essential Oils

**Figure 3:**
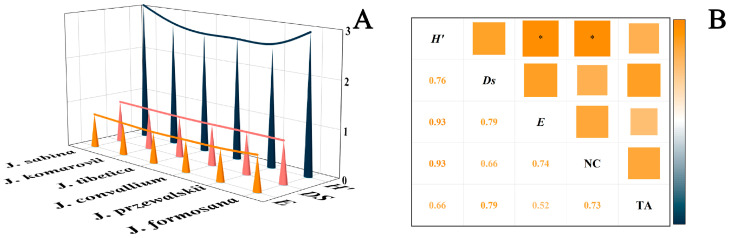
Chemical diversity (**A**) and correlation analysis (**B**) of essential oils from six *Juniperus* species. H′ is the Shannon–Wiener index; Ds is the Simpson’s diversity index; E is the Pielou evenness; NC is the number of compounds; TA is the total area;.“*”: *p* < 0.05.

To quantify the diversity of essential oil components, the α diversity index was introduced to calculate the richness and uniformity of components. There were differences in the α-diversity of EOs among different *Juniperus* plants (Figure 3A). The EOs from *J. sabina* had the highest α-diversity (Shannon–Wiener index: 3.06; Simpson’s variety index: 0.91; Pielou evenness: 0.74), and this meant a good variety and uniformity. *J. formosana* had the same Pielou evenness as *J. sabina*, with the Shannon–Wiener index (2.92) and Simpson’s variety index (0.89) ranked second, only below *J. sabina*. *J. przewalskii* oil had the least variety and homogeneity, with α-diversity indices of 2.50, 0.89, and 0.66, respectively.

The relationships between the chemical diversity of the EOs are shown in Figure 3B. Chemical diversity indices had a high degree of relevance to each other, and all of them were positively correlated. Results indicated that Shannon–Wiener index was highly correlated with the Pielou evenness and the number of compounds with the same maximum correlation coefficient of 0.93. Meanwhile, the correlations reached significant levels (Figure 3B).

#### 2.1.4. Chemometrics Analysis of *Juniperus* Essential Oils

**Figure 4:**
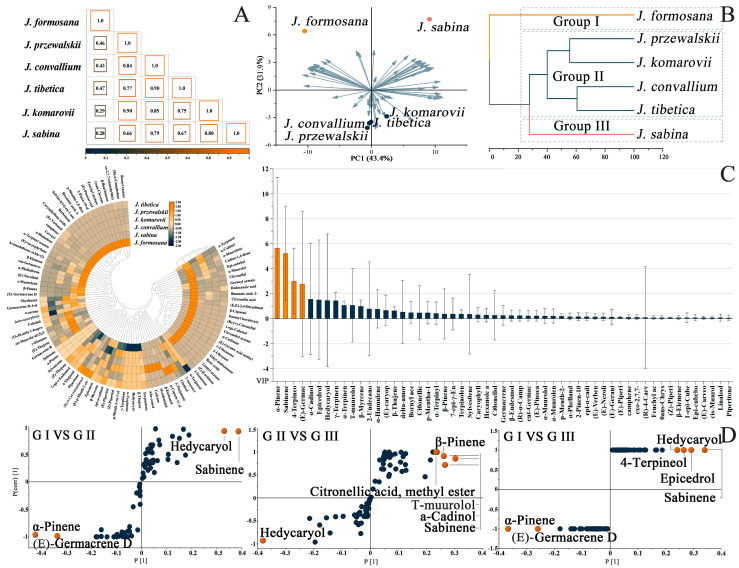
Correlation analysis (**A**), principal component analysis (PCA) and hierarchical clustering analysis (HCA) (**B**), correlation analysis and OPLS-DA (**C**), and S-plot (**D**) of essential oils from six *Juniperus* species. (The mark in orange is the chemical components with VIP values greater than 2).

The chemical profiles of the six oils showed positive connections, with the largest correlations of 0.90 occurring between *J. convallium* and *J. tibetica*, and *J. przewalskii* and *J. komarovii* (Figure 4A). The correlation between *J. formosana* and *J. sabina* was the weakest of 0.28. And the results of principal component analysis and cluster analysis were consistent with those of the correlation analysis employing the EOs constituents of six different species (Figure 4B), and they grouped *J. formosana* and *J. sabina* into clusters with more distant chemical relatives. The *X*-axis and *Y*-axis combined to account for 75.30% of the overall variability, which indicated that 92 components could well distinguished the six *Juniperus* plants.

Correlation analyses of essential oil components between different *Juniperus* plants were conducted, and the results showed that the isolation of *J. formosana* and *J. sabina* may be due to the different levels of some compounds (Figure 4C). Bornyl acetate, (R)-α-campholene aldehyde, exo-2,7,7-trimethylbicyclo [2.2.1] heptan-2-ol, β-bourbonene, (E)-chrysanthenyl acetate, fenchyl acetate, (E)-pinocarveol, 2-pinen-10-ol, etc., caused *J. formosana* to be distant from other plants, while EO ingredients, such as α-terpineol, α-cadinol, α-muurolene, cadine-1,4-diene, epi-cubebol, and α-muurolol, supported the independence of *J. sabina*. Some of these were unique components, hinting that the ingredients may play an important role in the chemical diversity of essential oils. And the compounds that stood out on the coefficient plot (Figure 4C) were α-pinene, sabinene, 4-terpineol, and (E)-germacrene D with VIP (variable important in projection) values greater than 2. These four components were the significant influence compounds in *Juniperus*, whose concentrations varied substantially, and could be considered as candidate markers to determine the chemotype of *Juniperus* EOs.

Figure 4D shows three different comparison models with selected candidate marker compounds. Group I contained *J. formosana* due to the high concentration of α-pinene (17.31%) and (E)-germacrene D (7.32%). The Group II cluster accumulated the most similar spectra. The main elements of the hedycaryol-rich type that formed Group II were hedycaryol (6.80–9.83%) and sabinene (12.14–17.50%), confirming the chemical similarity between *J. przewalskii*, *J. komarovii*, *J. convallium*, and *J. tibetica*. Finally, the richness of sabinene (19.83%) was a specific trait for Group III, which varied from the other two groups as a result of the abundant distinctive components.

### 2.2. Biological Activity of Juniperus Essential Oils

#### 2.2.1. Antioxidant Activity

DPPH and ABTS radical scavenging methods were used to detect the antioxidant activity of essential oils, and all essential oils exhibited antioxidant activity (Table 2). The IC50 of all extracts, as assessed by DPPH radical scavenging ability, ranged from 11.94 mg/mL to 45.62 mg/mL. The highest antioxidant activity was demonstrated by *J. komarovii*, whereas *J. przewalskii* displayed the lowest. Furthermore, the ABTS scavenging activity declined in the following order: *J. sabina* > *J. komarovii* > *J. formosana* > *J. convallium* > *J. tibetica* > *J. przewalskii*. Except for *J. przewalskii*, the other five plants had a stronger scavenging ability without exhibiting significant differences (*p* < 0.05).

#### 2.2.2. Antibacterial Activity

**Figure 5:**
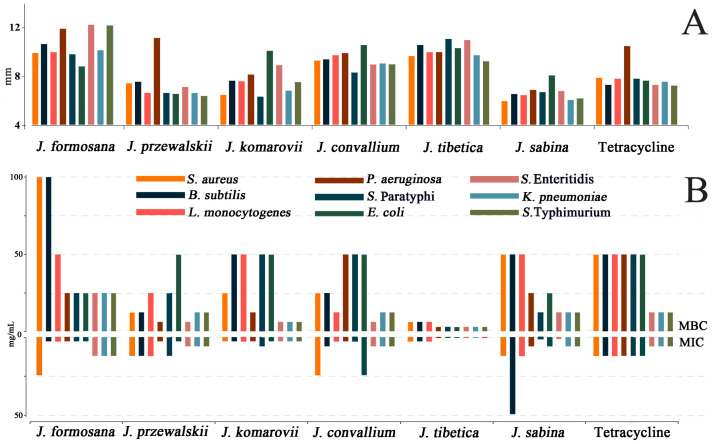
Inhibition zone diameter (**A**), and MIC and MBC (**B**) of the essential oils from six *Juniperus* species against nine bacteria.

As depicted in Figure 5A, the disc diffusion technique was used to assess the antibacterial activity of the EOs against nine pathogens. All of the examined microorganisms had varying degrees of susceptibility to the six EOs, with halos ranging from 6.10 to 12.25 mm. The preliminary screening of diameter inhibition data revealed that all EOs considerably reduced the pathogen growth to different degrees.

By assessing the MIC and MBC at various concentrations (0.39–400.00 mg/mL), the microdilution assay was used to further assess the antibacterial activity. With MIC values ranging from 0.78 to 50.00 mg/mL, all of the EOs showed broad-spectrum antibacterial activity against nine pathogenic bacteria in Figure 5B. As expected, the MBCs, which ranged from 3.13 to 100.00 mg/mL, were greater than the MICs. The EOs of *J. komarovii* produced the lowest MIC values (ranging from 0.78 to 3.13 mg/mL) and MBC values (ranging from 3.13 to 6.25 mg/mL), confirming that it was the most effective against Gram-negative bacteria. The MIC values (3.13–12.50 mg/mL) and MBC values (ranging from 6.25 to 50.00 mg/mL) of the EOs from *J. convallium* and *J. przewalskii* were both lower than the other three species. Moreover, *Salmonella* Enteritidis had the lowest MBC value and was susceptible to the six EOs.

### 2.3. Correlation Analysis of Compounds and Bioactivity

#### 2.3.1. Key Compounds Responsible for Antioxidant Activity

**Figure 6:**
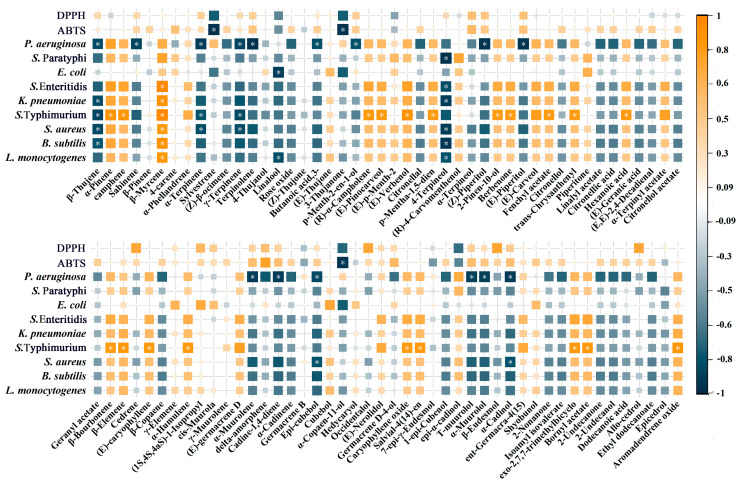
Correlation analysis of EOs compounds and bioactivity.

The correlation analysis was obtained using the volatile components of the six *Juniperus* oils as the X variables and the antioxidant data as the Y variables (DPPH values were the reciprocal values of IC_50_) to find the volatile substances associated with the antioxidant activity of the EOs (Figure 6). As a result of this analysis, all 92 compounds showed a correlation with the antioxidant activity of EOs. Despite the concentration of these substances fluctuates greatly, terpinolene, α-terpineol, (Z)-piperitol, cedrene, α-Muurolene, δ-amorphene, etc., were found to be favorably linked with antioxidant activity; while sylvestrene, linalool, 3-thujanone, α-copaen-11-ol, and epi-α-cadinol (T-cadinol) were inversely correlated with antioxidant activity. Due to the various scavenging mechanisms, essential oil components were more strongly correlated with ABTS than DPPH. The results indicated that sylvestrene, 3-thujanone, and α-copaen-11-ol were significantly correlated with ABTS, while the correlation coefficients of −0.96, −0.96, and −0.95 were found, respectively.

#### 2.3.2. Key Compounds Responsible for Antibacterial Activity

The diverse biological actions of EOs had been blamed for the components’ mosaic combination. The bacteriostatic effects of EOs differed with components or strains, as seen in Figure 6. The compounds 4-terpineol, β-myrcene, β-thujene, γ-terpinene, and epi-cubebol exhibited the widest spectrum of growth inhibition, and their contents were strongly correlated with the growth inhibition of tested bacteria. *Salmonella* Enteritidis and *Salmonella* Typhimurium have correlation coefficients of 0.97 and 0.98 with β-myrcene, respectively, showing the importance of β-myrcene in antibacterial action.

In the tested bacteria, *Pseudomonas aeruginosa*, *Salmonella* Typhimurium, and *Staphylococcus aureus* proved to be the most sensitive to EOs, compared with the weakest of *Salmonella* Paratyphi, and *Escherichia coli*. β-thujene, γ-terpinene, terpinolene, 4-terpineol, epi-cubebol, cadine-1,4-diene, α-cadinol, and other monoterpenes and its oxides were strongly connected with the inhibition of bacterial growth, particularly *Pseudomonas aeruginosa*, *Salmonella* Typhimurium, and *Staphylococcus aureus*. Among them, the content of monoterpenes and their oxides had a high degree of relevance to bacteriostatic activity, such as α-pinene, camphene, β-myrcene, (E)-thujone, (E)-p-menth-2-en-1-ol, (E)-verbenol, and (R)-4-Carvomenthenol, all of which were positively correlated with the inhibitory activity against the tested strains.

## 3. Discussion

Essential oils have the characteristics of complex composition and volatility, and the yield of various plant essential oils is affected by conditions including plant species, raw material origin, extraction method, and others [13,19,20,21,22,23]. The yields of six essential oils were significantly higher than the yields reported in some earlier literature (0.06–1.48%) [14,24,25,26,27]. In contrast, the highest extraction rate (6.55%) was found for the EOs extracted by supercritical extraction from *J. communis* [28].

In fact, as EOs are secondary components and are strongly influenced by environmental conditions, the distribution of essential oil components varies between examined samples [29]. Of the six *Juniperus* species, the largest number of chemical constituents of 63 was identified in *J. sabina* with the highest chemical diversity, but also less than the 82 of Adams [30]. In Adams [30,31], the oil from *J. przewalskii* (73), *J. convallium* (69), *J. tibetica* (82), and *J. komarovii* (62) was identified to contain more components than our study. This may be related to the unique natural environment of the Tibetan Plateau. In this environment, pressures such as low temperatures, wind blowing, and high-altitude ultraviolet radiation can accelerate the oxidation or hydrolysis of terpenes to other compounds, which had a polyartious effect on the content of secondary metabolites [22,32,33].

Each essential oil is characterized by some major compounds which can reach high levels, as compared to other compounds [34]. Monoterpenes, the major volatile components, are the typical characteristic of most *Juniperus* species [4,10,28,30,35]. In the present study, sabinene was also the most abundant ingredient in *J. tibetica*, *J. komarovii*, and *J. sabina*, and α-pinene predominated in *J. formosana*, in agreement with the literature data [10,36,37,38]. However, limonene and a-pinene served as major components of *J. convallium* and *J. przewalskii*, which differs from our findings [36,37,38].

Moreover, the amounts of main compounds widely varied between the examined samples. Abdel-Kader et al. (2019) showed the dominance of sabinene in *J. sabina*, which was relatively high (55.82%), significantly higher than the portion in our study (19.83%). And some compounds with rich content in other plants, like piperitone, were in low amounts or even not detected in our study [31,38]. Various studies [32,39] proved that populations of the same species collected from different habitat had a diverse essential oil composition. Natural factors may be at the root of the variability in the chemical composition of these essential oils, leading to the establishment of different chemical races or chemotypes within the same species and consequent changes in quality.

Through GC-MS analysis, 15 common compounds were identified in this study. Combining this study with past findings [24,40], α-pinene is the merely sole common component found in all *Juniperus* essential oil studies, indicating a higher variability in EOs composition in these taxa. The remaining 14 compounds are common and unique components of the six plants in this study. Part of these common and unique elements could be attributed to some species being endemic to China and partly to special ecological environments. And this also hinted at the similarity and polymorphism of the EOs compositions from different *Juniperus* species and different habitats.

The unique plateau environment may contribute to the discovery of some components, including (E)-geranic acid methyl ester, ethyl dodecanoate, rose oxide, isoamyl isovalerate, and β-copaene, which were first reported in *Juniperus*. The binding of 12 different compounds in *J. sabina* EOs also supported the specificity of EOs between species. Except for hedycaryol, these compounds are mainly trace components and may contribute significantly to the distinct differences in the scent and biological activity of the plants [41]. Hedycaryol, the content of which was determined to be 0.8–9.83%, is an important biosynthetic intermediate toward eudesmols and guaiols, which showed potent insecticidal action against agricultural pests and may be an advantageous and eco-friendly biopesticide [42,43].

The composition and profile of EOs is highly varied between different individuals of *Juniperus* and it is thus that these individuals can be definite as chemotypes. A number of chemotypes have been recorded in earlier research, including the prevalence of α-pinene, β-pinene, sabinene, β-phellandrene, limonene, δ-3-carene, β-thujone, and manoyl oxide [44,45,46]. In our study, the hedycaryol chemotype was defined in *Juniperus* for the first time, while *J. sabina* belongs to this group because of its different chemical characteristics. However, whether the presence of hedycaryol chemotype in the EOs from *Juniperus* needles is a trait that can be used for taxonomic purposes requires more extensive chemical investigation of the genus.

*Juniperus* were first established by Linnaeus in 1753. The 1978 Flora Reipublicae Popularis Sinicae split *Juniperus* into *Juniperus* and *Sabina* (Editorial Committee of FRPS), while the 1999 English Flora of China incorporated *Sabina* into *Juniperus* [47]. Thus, the division and incorporation of the *Juniperus* has been debated for a long time. Using RAPD data, Adams (1993) separated the genus into three genera of *Juniperus*, *Sabina*, and *Caryocedrus*. In this study, the low similarity between *J. sabina* and the other five species provides a chemical basis for the division between *Juniperus* and *Sabina*. Previous studies have also shown consistency between chemical and genetic diversity [33,48,49].

The fact that *J. formosana* was distantly linked to the four species of Group II species was incredible. Previous studies have shown that the role of phenotypic plasticity in chemical composition cannot be ignored [50,51]. Morphologically, *J. formosana* leaves are spiny, while the other five species are scaly leaves or a mixture of scaly and spiny leaves. Another possible reason for this was the low altitude of the species sampling site. The variety of plant essential oils can vary depending on small habitat differences between altitudes [52]. Thus, it is crucial to research the chemical composition and biological properties of various *Juniperus* species’ EOs for its systematic taxonomic study, which also needs further research.

Plant essential oils have direct or indirect antioxidant activity. The six EOs appeared to provide positive health benefits for consumers, according to the DPPH and ABTS values. And first discovered as an antioxidant, *J. przewalskii*, *J. convallium*, *J. tibetica*, and *J. komarovii* EOs open up new possibilities for the use of *Juniperus* needles and the creation of natural plant remedies. This activity was attributed to camphor, camphene, and other components like δ-amorphene, β-eudesmol, and α-muurolol.

Among the six species, the antioxidant activity of *J. komarovii* and *J. sabina* was prominent. Their antioxidant differences with *J. przewalskii* were not surprising. Previous studies have shown that hydroxylated compounds and terpenes can partially account for the inhibitory effect of EOs [53]. Hydroxylated compounds exhibited a variety of chemical properties and reactivity by trapping radical-to-organic radical reagents (DPPH and ABTS tests) [54], and the process was influenced by the degree of hydroxylation, extent of conjugation, and fundamental interaction [54]. Terpenes break conjugated double bonds and release small and high-affinity hydrogen radicals to neutralize oxygen radicals and reduce oxidative damage. Therefore, their different antioxidant efficiency results from the presence of phenolic compounds and terpenes with conjugated double bonds, which act as donors of hydrogen and electrons, and from their different concentration levels in these natural mixtures.

The correlation analysis supported the evidence that compounds in the greatest proportions may not necessarily be responsible for the largest share of the antioxidant activity, while less abundant constituents with strong therapeutic effects may be helpful in boosting the antioxidant activity of EOs. Phenolic compounds, for example, whose hydroxyl groups donate hydrogen atoms, play a significant role in free radical elimination and bioactive potential [55]. The loss of the allylic hydrogen atom is linked to the neutralization of the DPPH and ABTS radicals by terpenes with conjugated double bonds, like β-pinene, β-eudesmol, (E)-germacrene D, citronellal, and so on [56]. However, it must be highlighted that the mechanisms behind the antioxidant action of plant secondary metabolites are complex and incompletely known; further study is required to fully grasp the underlying chemical route.

*Juniperus* EOs had bacteriostatic effects on all of the studied strains, and except for *J. formosana*, the antibacterial activity of the other five species was the first measured. Overall, it appeared to be more effective against Gram-negative bacteria than Gram-positive bacteria. On the contrary, due to different cell wall compositions, Gram-positive bacteria were more sensitive to plant EOs in earlier investigations [57,58]. This may be due to the high concentration of oxygenated monoterpene and the special compositions that are more sensitive to the cell wall of Gram-negative bacteria and enable them to partition in the lipids of the cell wall, disturb the cell structures, and increase the permeability [59,60]. Death may result from significant leakage from bacterial cells or the escape of essential chemicals and ions.

As EOs are mixtures of numerous components, their antibacterial activity generally derives from specific components configuration and interactions between components [61]. Phenols and aldehydes showed higher antibacterial capacities among the ingredients of EOs, followed by alcohols, ketones, esters, and hydrocarbons [62]. By establishing hydrogen bonds with the active sites of the target enzymes and inactivating them, the hydroxyl groups found in phenolic compounds were extremely effective against a variety of bacteria [63]. Certain phenols possess antibacterial effects even at extremely low concentrations [64]. From the perspective of three chemotypes, hedycaryol-rich EOs appeared to have stronger antibacterial capacity.

Due to the difference in concentration, monoterpenes and their oxides, such as 4-terpineol, β-myrcene, β-thujene, and γ-terpinene, appeared to have a larger bacteriostatic effect than sesquiterpenes and their oxides. And this seems to provide viable objectives for additional investigation to pinpoint the active EOs’ antibacterial action. Of course, it is possible that different ingredients in the EOs present a synergistic interaction against bacteria. According to Leandro et al. [65], the functionality of the chemicals found in EOs should not be attributed in isolation, but rather additively, synergistically, or antagonistically. Even yet, the connection and correlation coefficient between the components of EOs and their antibacterial activity offered guidance and foundation for additional study to substantiate their antibacterial mechanism. In conclusion, EOs are potential agents against Gram-positive and Gram-negative bacteria [66]. Similar research can explore the possible function of EOs as antibacterial agents, but more research is necessary.

## 4. Materials and Methods

### 4.1. Plant Material and the Extraction of EOs

The needles were gathered from the Tibetan Plateau, and Table 3 summarizes the background details of the samples. From each patch, five individual plants were randomly selected: healthy and complete needles were obtained from four different directions on each tree and then evenly mixed. The species were identified using morphological characteristics and information found in the library of the School of Forestry at Northwest A & F University, Yangling, China. Voucher specimens were placed at the Shaanxi Key Laboratory of Economic Plant Resources Development and Utilization. The needles were air dried before being processed into powders. According to Zhang et al. [67] with some modifications, the hydro-distillation of sample powders was carried out for 5 h using a modified Clevenger type equipment to obtain EOs. Following separation, the oils were dried in anhydrous sodium sulfate. We produced three replications for EOs extractions from each specie and mixed them for homogenization. Pure EOs weights and volumes were measured, and they were then sealed in brown glass vials and kept in a −20 °C freezer until further analysis. A dry weight basis was used to calculate the oil yields (*w*/*w*).

### 4.2. GC-MS Analysis

TG-5MS capillary column-equipped TRACE1310-ISQLT equipment (Thermo Fisher Scientific, Wyman Street, Waltham, MA, USA) was used for the analysis. Helium (purity 99.999%) was the carrier gas at a flow rate of 1 mL/min with an ionization voltage of 70 eV, covering a mass range 40–460 *m*/*z*. The temperature of the GC oven was kept at 35 °C for 3 min, increased to 150 °C at 3 °C/min, then ramped up to 260 °C at 10 °C/min, before being held at 290 °C at 5 °C/min for 8 min. The constituents were identified by comparing constituents’ mass spectra with the NIST 08, C8-C40 n-alkane standard solution and published mass spectra [35]. The peak area normalization was used to determine the relative concentrations of the components.

### 4.3. Chemodiversity

Ecologically, species diversity is divided into α, β, and γ diversity according to various research scales [68]. The Shannon–Wiener index, Simpson diversity index, and Pielou evenness are all used to calculate α-diversity, which primarily focuses on the number of species in limited homogeneous ecosystems [69]. It is also referred to as the diversity inside the biological region or species diversity within a single sample, separated from other samples. Hence, α-diversity could be utilized to calculate the chemical diversity of EOs [52]. Similarly, the Shannon–Wiener index and Pielou evenness could be used to gauge the evenness of EOs composition, and the Shannon diversity index can gauge the richness of its composition.

$$H\prime =-\sum P_{i}\ln P_{i}$$

$$D_{S}=1-\sum P_{i}^{2}$$

$$E=\frac{H\prime }{\ln S}$$

where *H*′ is the Shannon–Wiener index; *D_S_* is the Simpson’s diversity index; *E* is the Pielou evenness; *P_i_* is the proportion of compounds in the sample; *S* is the number of compounds.

### 4.4. Antioxidant Activity

#### 4.4.1. DPPH

There are several methods for assessing antioxidant activity that rely on specific substrates and mechanisms [70]. In a modified assay, 2 mL of 0.1 mM DPPH radical solution in 80% ethanol was mixed with 2 mL of various concentrations of the EOs. The absorbance was measured at 517 nm using an ultraviolet visible spectrophotometer (UV-1780, Shimadzu Corporation, Kyoto, Japan) against the control. The percentage scavenging was then plotted against the concentration and a regression equation was obtained to calculate the IC_50_ (mg/mL) (concentration of the EOs that caused 50% of DPPH radical scavenging).

#### 4.4.2. ABTS

The antioxidant activity was measured using ABTS’s modified technique [71]. The absorbance of 3.90 mL of diluted ABTS solution and 0.10 mL of EOs was measured at 734 nm after being incubated at 37 °C for 10 min. To generate a standard curve, a trolox standard solution (0 to 800 µmol/L) was used. The trolox equivalent in micromoles per gram was used to express the inhibitory capacity of ABTS.

### 4.5. Antibacterial Activity

#### 4.5.1. Antimicrobial Strains

The bacteria were supplied by the Microbial Culture Collection Center of the Guangdong Institute of Microbiology in China. The six Gram-negative bacteria were *Salmonella* Paratyphi (CMCC50093), *Escherichia coli* (ATCC25922), *Salmonella* Typhimurium (CMCC50115), *Salmonella* Enteritidis (ATCC14028), *Pseudomonas aeruginosa* (ATCC27853), and *Klebsiella pneumoniae* (ATCC46117). The three Gram-positive bacteria were *Staphylococcus aureus* (ATCC25923), *Listeria monocytogenes* (ATCC19115), and *Bacillus subtilis* (ATCC6633). The bacteria were revived using two subcultures in Mueller–Hinton broth, and a suspension of the bacteria in sterile peptone water was prepared and adjusted to the mid-exponential growth phase with an optical density of 0.5 at 600 nm.

#### 4.5.2. Disc Diffusion Method

The antibacterial activity was evaluated by the disc diffusion method using Müeller–Hinton agar described by Meng et al. [72] with some modification. An aliquot of soft agar was prepared and 0.1 mL bacterial suspension were added over each plate (diameter 90 mm) containing 25 mL nutrient medium. The EOs were prepared as a 100 mg/mL solution with 1% dimethyl sulfoxide (DMSO). Sterile paper discs (6 mm) were impregnated with 100 mg/mL EOs for 2 h and placed on the inoculated agar. After 24 h of incubation at 37 °C, the antibacterial activity was estimated by measuring the diameter of bacterial growth inhibition zones (mm) around discs. Positive and negative controls were 1% DMSO solution and tetracycline (10 μg/mL), respectively.

#### 4.5.3. Determination of the Minimum Inhibitory Concentration and Minimal Bactericidal Concentration

The minimum inhibitory concentration (MIC) and minimal bactericidal concentration (MBC) of EOs was determined by the reduced half dilution method in 96-well plates. Mueller–Hinton broth medium was mixed with EOs solutions at different concentrations so as to obtain samples with the final concentrations of 0.195–100 μg/mL [67,73]. All samples were incubated with bacteria for 24 h at 37 °C, and the inoculum and culture medium served as the positive and negative control [74,75]. MIC was defined as the lowest concentration of essential oils inhibiting visible bacterial growth. Microorganism culture medium showing no turbidity was transferred to agar plates and cultured at 37 °C for 24 h. The corresponding concentration without visual bacteria growth was taken as the minimal bactericide concentration (MBC).

### 4.6. Statistical Analysis

Each experiment was performed 3–5 times. Using the Statistical Package for the Social Science, a one-way analysis of variance was used to determine the statistical significance between the test groups (SPSS 18.0, SPSS Inc., Chicago, IL, USA). Multivariate analyses, including upset analysis, hierarchical cluster analysis, and correlation analysis were carried out using R, SIMCA 13.0, and Origin 2022. Findings were regarded as statistically significant if *p* < 0.05.

## 5. Conclusions

The geographical location and climatic conditions of Qinghai-Tibet Plateau have produced the unique characteristics of EOs from *Juniperus*. The six *Juniperus* essential oils have a rich chemodiversity and there are great differences among species with the most outstanding being *J. sabina*. The chemodiversity of the essential oils of *Juniperus* species provides a chemical basis for the taxonomy of *Juniperus*. *J. convallium*, *J. komarovii*, and *J. sabina* showed a strong antioxidant activity, while *J. formosana*, *J. tibetica*, and *J. convallium* supplied the tested bacteria with distinctive antibacterial activities, indicating that they could be used to create natural antioxidants and antibiotics.

## Figures and Tables

**Table 1:** Relative contents of EOs compounds of six *Juniperus* species.

NO.	Compounds	RI ^a^	RI ^b^	Area (%)					
				*J. formosana*	*J. przewalskii*	*J. convallium*	*J. tibetica*	*J. komarovii*	*J.* *sabina*
Monoterpene hydrocarbons									
**1**	β-Thujene	920	902	0.16	1.28	1.04	1.09	1.93	1.76
**2**	α-Pinene	940	911	17.31	5.86	5.58	6.93	2.71	2.74
**3**	Camphene	954	927	0.32	0.04	0.05	0.07	0.03	0.04
**4**	Sabinene	975	956	4.91	12.14	15.33	16.13	17.5	19.83
**5**	β-Pinene	980	959	2.15	0.06	0.05	0.07	nd	2.63
**6**	β-Myrcene	991	973	3.13	0.53	1.38	1.85	1.17	nd
**7**	4-Carene	1001	981	0.38	nd	0.37	0.83	0.06	0.92
**8**	α-Phellandrene	1002	985	0.58	0.04	0.02	0.04	0.12	0.19
**9**	α-Terpinene	1007	996	0.29	1.78	1.54	2	3.19	3.15
**10**	Sylvestrene	1027	1009	3.49	8.58	2.35	3.78	3.07	1.84
**11**	(Z)-β-Ocimene	1031	1020	0.01	nd	nd	tr	nd	0.15
**12**	γ-Terpinene	1054	1042	0.56	2.99	2.46	3.02	4.42	4.46
**13**	Terpinolene	1086	1072	0.62	0.9	0.89	1.18	1.48	1.8
Oxygenated monoterpenes									
**14**	4-Thujanol	1077	1051	nd	0.18	0.25	0.21	0.2	0.14
**15**	Linalool	1095	1083	0.21	0.79	nd	0.13	0.17	0.68
**16**	Rose oxide	1108	1089	nd	nd	nd	nd	nd	0.12
**17**	(Z)-Thujone	1111	1091	nd	0.18	0.02	0.25	0.22	0.03
**18**	Butanoic acid, 3-methyl-, 3-methyl-3-butenyl ester	1112	1096	nd	0.01	nd	nd	0.05	0.23
**19**	(E)-Thujone	1114	1097	0.02	nd	0.11	nd	nd	nd
**20**	3-Thujanone	1124	1099	nd	0.9	nd	nd	nd	nd
**21**	p-Menth-2-en-1-ol	1125	1102	0.06	0.28	0.33	0.6	0.62	0.59
**22**	(R)-α-Campholene aldehyde	1126	1107	0.63	nd	0.02	0.03	nd	nd
**23**	(E)-Pinocarveol	1135	1121	0.57	nd	nd	nd	nd	nd
**24**	(E)-p-Menth-2-en-1-ol	1140	1123	nd	0.2	0.23	0.44	nd	nd
**25**	(E)-Verbenol	1144	1128	0.39	nd	0.03	0.05	nd	nd
**26**	Citronellal	1152	1139	nd	tr	nd	nd	nd	0.32
**27**	p-Mentha-1,5-dien-8-ol	1170	1150	1.2	nd	nd	nd	nd	nd
**28**	4-Terpineol	1177	1161	1.16	6.58	5.55	nd	8.81	8.96
**29**	(R)-4-Carvomenthenol	1182	1163	nd	nd	nd	6.55	nd	nd
**30**	α-Terpineol	1186	1175	0.42	0.2	0.16	0.19	0.5	0.58
**31**	(Z)-Piperitol	1195	1179	nd	0.07	0.07	0.14	0.22	0.24
**32**	2-Pinen-10-ol	1198	1179	0.41	nd	nd	nd	nd	nd
**33**	Berbenone	1204	1191	0.16	nd	nd	nd	nd	nd
**34**	(E)-Piperitol	1207	1192	nd	0.09	0.11	0.21	0.32	0.35
**35**	(E)-Carveol	1215	1200	0.2	0.02	tr	0.01	nd	nd
**36**	Fenchyl acetate	1223	1202	0.22	nd	nd	nd	nd	nd
**37**	Citronellol	1228	1210	0.38	0.06	0.01	0.03	0.04	2.31
**38**	(E)-Chrysanthenyl acetate	1238	1217	0.22	nd	nd	nd	nd	nd
**39**	Piperitone	1249	1239	nd	0.01	0.49	0.89	0.01	0.37
**40**	Linalyl acetate	1254	1241	nd	nd	nd	nd	nd	0.29
**41**	Citronellic acid, methyl ester	1261	1244	nd	nd	0.01	0.01	0.11	2.55
**42**	Hexanoic acid, 3-methyl-2-butenyl ester	1292	1277	0.74	nd	nd	nd	nd	nd
**43**	(E)-Geranic acid methyl ester	1315	1306	nd	nd	nd	nd	nd	0.72
**44**	(E,E)-2,4-Decadienal	1315	1297	0.01	nd	0.01	0.01	nd	0.13
**45**	α-Terpinyl acetate	1349	1334	1.12	nd	nd	nd	nd	0.1
**46**	Citronellol acetate	1350	1337	nd	nd	nd	nd	nd	0.28
**47**	Geranyl acetate	1379	1366	0.05	nd	nd	nd	nd	0.18
Sesquiterpene hydrocarbons									
**48**	β-Bourbonene	1388	1372	0.14	nd	nd	nd	nd	nd
**49**	β-Elemene	1390	1378	0.25	nd	0.12	0.03	0.01	0.06
**50**	Cedrene	1413	1392	nd	nd	nd	tr	0.11	nd
**51**	(E)-Caryophyllene	1417	1407	1.77	0.14	0.23	0.05	nd	0.1
**52**	β-Copaene	1430	1411	nd	nd	nd	nd	nd	0.02
**53**	γ-Elemene	1434	1419	nd	nd	0.3	0.04	0.04	0.04
**54**	α-Humulene	1454	1442	2.01	nd	0.05	0.01	0.02	0.08
**55**	(1S,4S,4aS)-1-Isopropyl-4,7-dimethyl-1,2,3,4,4a,5-hexahydronaphthalene	1458	1461	nd	nd	0.16	0.07	0.13	nd
**56**	(Z)-Muurola-4(15),5-diene	1465	1464	nd	nd	0.57	nd	0.19	0.12
**57**	γ-Muurolene	1478	1464	0.28	nd	nd	nd	nd	0.23
**58**	(E)-Germacrene D	1485	1470	7.32	0.04	nd	0.06	nd	0.58
**59**	α-Muurolene	1500	1483	nd	nd	nd	0.14	0.37	0.83
**60**	δ-Amorphene	1522	1502	1.28	nd	2.09	1.03	1.72	3.76
**61**	Cadine-1,4-diene	1533	1509	nd	nd	nd	0.01	0.06	0.13
**62**	α-Cadinene	1538	1520	nd	nd	nd	nd	nd	0.22
**63**	Germacrene B	1559	1530	nd	0.4	0.68	nd	nd	0.05
Oxygenated sesquiterpenes									
**64**	Epi-cubebol	1493	1482	nd	0.03	nd	nd	0.12	0.32
**65**	Cubebol	1514	1496	nd	nd	0.74	0.28	nd	nd
**66**	α-Copaen-11-ol	1539	1522	nd	0.19	nd	nd	0.01	nd
**67**	Hedycaryol	1548	1523	0.8	6.8	9.83	9.44	7.15	1
**68**	Occidentalol	1550	1528	nd	nd	nd	nd	0.13	nd
**69**	(E)-Nerolidol	1561	1534	0.48	nd	nd	nd	0.2	0.04
**70**	Germacrene D-4-ol	1575	1541	0.46	0.18	1.34	0.61	0.54	1.65
**71**	Caryophyllene oxide	1582	1547	0.79	0.01	nd	nd	nd	nd
**72**	Salvial-4(14)-en-1-one	1594	1553	0.12	nd	nd	nd	nd	nd
**73**	7-epi-γ-Eudesmol	1622	1574	nd	2.14	2.07	1.62	1.62	nd
**74**	1-epi-Cubenol	1627	1577	nd	nd	nd	nd	nd	0.45
**75**	epi-α-Cadinol (T-cadinol)	1638	1579	0.39	0.48	nd	0.58	nd	nd
**76**	T-Muurolol	1640	1579	nd	nd	1	nd	1.65	4.11
**77**	α-Muurolol	1644	1581	nd	0.05	0.13	0.08	0.29	0.89
**78**	β-Eudesmol	1649	1587	nd	0.01	nd	nd	1.29	0.09
**79**	α-Cadinol	1652	1590	nd	nd	nd	nd	3.04	5.69
**80**	ent-Germacra-4(15),5,10(14)-trien-1β-ol	1685	1603	0.73	nd	nd	nd	0.12	0.16
**81**	Shyobunol	1688	1605	0.05	nd	0.1	0.04	nd	0.07
Others									
**82**	2-Nonanone	1087	1075	0.02	0.01	nd	nd	nd	0.21
**83**	Isoamyl isovalerate	1102	1085	nd	nd	nd	nd	nd	0.16
**84**	exo-2,7,7-trimethylbicyclo [2.2.1] heptan-2-ol	1146	1132	0.28	nd	nd	0.02	nd	nd
**85**	Bornyl acetate	1288	1271	1.28	0.02	0.08	0.1	0.05	0.04
**86**	2-Undecanone	1293	1279	nd	nd	nd	nd	nd	4.37
**87**	2-Undecanol	1301	1285	nd	nd	nd	nd	nd	0.25
**88**	Dodecanoic acid	1565	1540	nd	nd	nd	0.11	nd	0.4
**89**	Allo-cedrol	1589	1550	nd	nd	nd	nd	0.12	nd
**90**	Ethyl dodecanoate	1594	1550	nd	nd	nd	nd	nd	0.24
**91**	Epicedrol	1618	1559	1.18	7.76	0.32	0.81	10.48	nd
**92**	Aromadendrene oxide-(2)	1678	1600	0.18	nd	0.08	nd	nd	nd
93	Total monoterpenes			42.56	43.85	38.6	46.94	47.18	59.09
94	Monoterpene hydrocarbons			34.39	34.28	31.2	37.19	35.91	39.92
95	Oxygenated monoterpenes			8.17	9.57	7.4	9.75	11.27	19.17
96	Total sesquiterpenes			16.87	10.47	19.41	14.09	18.81	20.69
97	Sesquiterpene hydrocarbons			13.05	0.58	4.2	1.44	2.65	6.22
98	Oxygenated sesquiterpenes			3.82	9.89	15.21	12.65	16.16	14.47
99	Others			2.94	7.79	0.6	1.13	10.65	5.67
100	Total area (%)			61.89	62.03	58.35	61.87	76.41	85.04
101	Number of compounds			52	40	45	48	47	63
102	Yield			4.13%	3.40%	2.77%	2.53%	1.63%	1.30%

NO.: number. RI ^a^: retention index from the literature. RI ^b^: retention index calculated against n-alkanes. nd: not detected. tr: trace (<0.01%).

**Table 2:** Antioxidant activity of EOs from six *Juniperus* species.

NO.	Samples	DPPH (IC_50_) (mg/mL)	ABTS (µmol Trolox/g)
1	*J. formosana*	18.83 ± 0.90 ^cd^	44.34 ± 7.55 ^a^
2	*J. przewalskii*	45.62 ± 0.37 ^e^	25.10 ± 7.98 ^b^
3	*J. convallium*	16.89 ± 3.14 ^c^	44.19 ± 0.46 ^a^
4	*J. tibetica*	21.26 ± 2.19 ^d^	43.73 ± 1.62 ^a^
5	*J. komarovii*	11.94 ± 0.14 ^b^	48.83 ± 0.88 ^a^
6	*J. sabina*	17.82 ± 0.11 ^c^	49.34 ± 0.95 ^a^
7	Trolox	0.01 ± 0.21 ^a^	-

Data (means ± SD, n = 3) within a row with different superscripts are significantly different (*p* < 0.05).

**Table 3:** Detailed information of six *Juniperus* species.

No.	Herbarium No	Species	Collection Place	Coordinates	Height	Sample Plots
1	JF. 20. 24	*J. formosana*	Guanting Town, Minhe County, Qinghai Province, China	N 35.757222° E 102.434444°	2350 m	3
2	JP. 20. 37	*J. przewalskii*	Maixiu Forest Farm, Zeku County, Huangnan Tibetan Autonomous Prefecture, Qinghai Province, China	N 35.228333° E 101.851667°	3519 m	24
3	JC. 20. 39	*J. convallium*	Jiangxi Forest Farm, Yushu County, Yushu Prefecture, Qinghai Province, China	N 32.055833° E 97.0038889°	3520 m	3
4	JT. 20. 77	*J. tibetica*	Jiangxi Forest Farm, Yushu County, Yushu Prefecture, Qinghai Province, China	N 32.072777° E 97.0241667°	3600 m	21
5	JK. 20. 31	*J. komarovii*	Doke River Forest Farm, Banma County, Guoluo Tibetan Autonomous Prefecture, Qinghai Province, China	N 32.745833° E 100.751111°	3550 m	3
6	JS. 20. 24	*J. sabina*	Ketusha District, Haiyan County, Haibei Prefecture, Qinghai Province, China	N 36.759662° E 100.794524°	3317 m	3

## Data Availability

Not applicable.
